# Decade-long delayed diagnosis of hypophosphatasia until next generation tooth loss: case reports on dental rehabilitation, diagnostic challenges and clinical implications

**DOI:** 10.3389/froh.2025.1585188

**Published:** 2025-07-18

**Authors:** Yating Cao, Feng Zhang, Xingxing Huang, Ai Jiang, Shaoyun Jiang, Min Wu

**Affiliations:** ^1^ Department of Stomatology, Shenzhen Maternity and Child Healthcare Hospital, Southern Medical University, Shenzhen, Guangdong, China; ^2^ Stomatological Center, Peking University Shenzhen Hospital, Shenzhen, Guangdong, China

**Keywords:** delayed diagnosis, hypophosphatasia, tooth loss, dental rehabilitation, case report

## Abstract

Hypophosphatasia (HPP) is a rare metabolic bone disorder caused by functional mutations in the gene Alkaline Phosphatase, Liver/Bone/Kidney (*ALPL*), resulting in impaired mineralization of bones and teeth. We report a female patient who experienced adolescent teeth loss and underwent dental rehabilitation, yet remained undiagnosed until her son developed similar symptoms before age two. Genetic testing confirmed HPP in both cases, more than a decade after her initial symptoms appeared. After multidisciplinary dental treatment, the woman's oral condition has remained stable. This case is helpful for dental professionals to enhance their understanding of HPP, thereby reducing misdiagnosis and delayed diagnosis and further preventing the intergenerational transmission of the disease. It discusses the reasons for delayed diagnosis and misdiagnosis, as well as insights into diagnostic approaches, treatment strategies and clinical implications. It emphasizes the critical need for oral professionals to enhance their understanding of HPP and to apply clinical examination methods rationally to ensure timely and appropriate diagnosis and treatment.

## Introduction

Hypophosphatasia (HPP) is a rare metabolic bone disorder characterized by reduced serum tissue-nonspecific alkaline phosphatase (TNSALP) activity. It can be inherited as an autosomal dominant or recessive genetic disease and often leads to impaired mineralization of bones and teeth. HPP was first reported in 1948 by Rathbun as a new skeletal pathology, presenting with extremely low levels of alkaline phosphatase and seizures (1). It is now recognized as a rare heterogeneous genetic disorder affecting bone and mineral metabolism, caused by functional deficiency mutations in the gene Alkaline Phosphatase, Liver/Bone/Kidney (*ALPL*). To date, over 400 *ALPL* gene mutations have been identified in various ethnic backgrounds, predominantly consisting of missense mutations (https://alplmutationdatabase.jku.at).

Based on the age of onset, HPP can be categorized into several distinct types: perinatal lethal, prenatal benign, infantile, childhood, adult, and odontohypophosphatasia. Odontohypophosphatasia is a subtype that affects only the teeth without any other abnormalities. It is the mildest subtype and diagnosed according to dental disease and HPP's biochemical features. However, it should be noted that children with odontohypophosphatasia may develop into adult or childhood subtypes (2). HPP often presents with non-traumatic premature loss of teeth. Some cases have reported the phenomenon of enlarged pulp chambers.

The reported prevalence of HPP worldwide varies greatly. Earlier research reported a prevalence of severe HPP of 1 in 100,000 in Canada and 1 in 300,000 in Europe (3, 4). Model predictions suggest mild HPP affects 1 in 6,370 individuals (4). Recent studies found that the prevalence is 6.2 per 100,000 among children in southern Israel, and 10.5 per 100,000 among Bedouin children (5). However, an algorithm-based analysis estimated the prevalence of mild HPP to be 1 in 508 (6), indicating it may be more common than previously thought (7).

Due to the diversity of clinical presentations and overlapping symptoms of HPP, it is easy to be delayed diagnosis and misdiagnosis. In this study, we reported the diagnostic and treatment processes and the 2-year prognosis follow-up of this mother and son. Through these two cases, it is helpful to enhance the awareness of HPP among dental professionals, reduce misdiagnosis and delayed diagnosis, and thus avoid the intergenerational inheritance of the disease. In addition, the article also explored the factors to be considered in the diagnosis of HPP, dental screening methods, management strategies, and clinical implications.

## Case 1

A 26-year-old female, height 169 cm, without any physical abnormalities or systemic diseases came to our hospital on September 2020, with a main complaint of “periodontal abscess around tooth 36”. She reported dental mobility and subsequent teeth loss during her high school years at the age of 15. She subsequently underwent lower dental crown and bridge restoration. Her mother and her mother's siblings didn't have a history of premature tooth loss. She has lost contact with her father since 2 years old.

**Figure 1:**
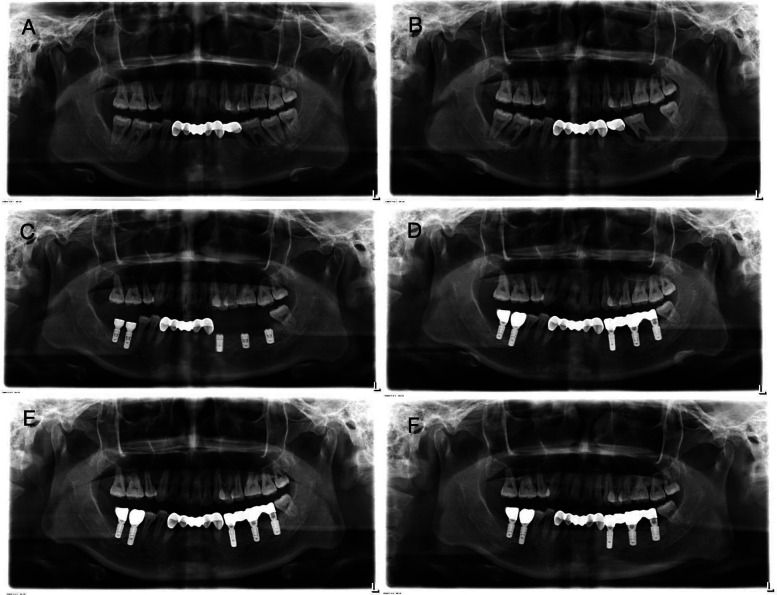
Panoramic view of case 1 patient. **(A)** First visit before pregnancy. The alveolar bone of tooth 36 was destroyed and absorbed to the apex, accompanied by the shadow of the root furcation. Significant radiolucent area surrounded the root apex of tooth 37. Bone resorption around tooth 46 was also present. **(B)** Four months after delivery. It revealed extensive radiolucency around the roots of teeth 36 and 46. **(C)** After implantation. **(D)** After implantation and restoration. **(E)** One month after implantation and restoration. **(F)** One year after implantation and restoration. The panoramic radiograph shows stable alveolar bone throughout the mouth.

The oral examination revealed that her oral hygiene was not particularly bad (Plaque Index = 0–1, Calculus Index = 0–2). Teeth 14, 31, 34, and 41 were found to be missing. All-ceramic crown and bridge restorations were performed on teeth 34–35 and teeth 33–43 and remained firm. A buccal periodontal abscess with fluctuating sensation was detected near the root apex of tooth 36. The mobility of tooth 36 and 37 was grade III and II, respectively. Based on the intraoral examination and panoramic radiograph revealing extensive bone loss affecting multiple teeth (Figure 1A), in the absence of significant systemic medical history, she was diagnosed with “periodontitis”.

After undergoing the first round of periodontal therapy, periodontal abscess disappeared. Subsequently, due to pregnancy, she re-examined four months after childbirth at the age of 28 in 2022 and tooth 37 was lost a week ago. Her antenatal check-ups during pregnancy did not reveal any abnormalities. Teeth 36 and 46 exhibited grade III mobility. A deep probing depth was found in tooth 47, which exhibited grade II mobility. Tooth 35 also exhibited grade II mobility. The panoramic x-ray was taken again as shown in Figure 1B, revealing extensive radiolucency around the roots of teeth 36 and 46. Due to severe bone defects and the need for implant restoration, teeth 35, 36, 46 and 47 have been extracted. The remaining teeth underwent periodontal treatment.

Four months after extraction, Straumann® implants (Institut Straumann AG, Basel, Switzerland) were placed at sites 46 (4.1 × 10 mm SLA BL), 47 (4.1 × 8 mm SLA BL), 34 (3.3 × 10 mm *Roxolid*® BL), 36 (4.1 × 8 mm SLA BL), and 37 (4.8 × 8 mm SLA BL). Simultaneous GBR was performed using Geistlich Bio-Oss® spongious bone substitute granules (0.25–1 mm particle size, Geistlich Pharma AG, Wolhusen, Switzerland) and Geistlich Bio-Gide® collagen membranes (25 × 25 mm). 34, 36, and 37 were for submerged healing, while 46 and 47 underwent transmucosal healing. Panoramic radiographs related to the dental implant procedure are shown below (Figures 1C–E).

**Figure 2:**
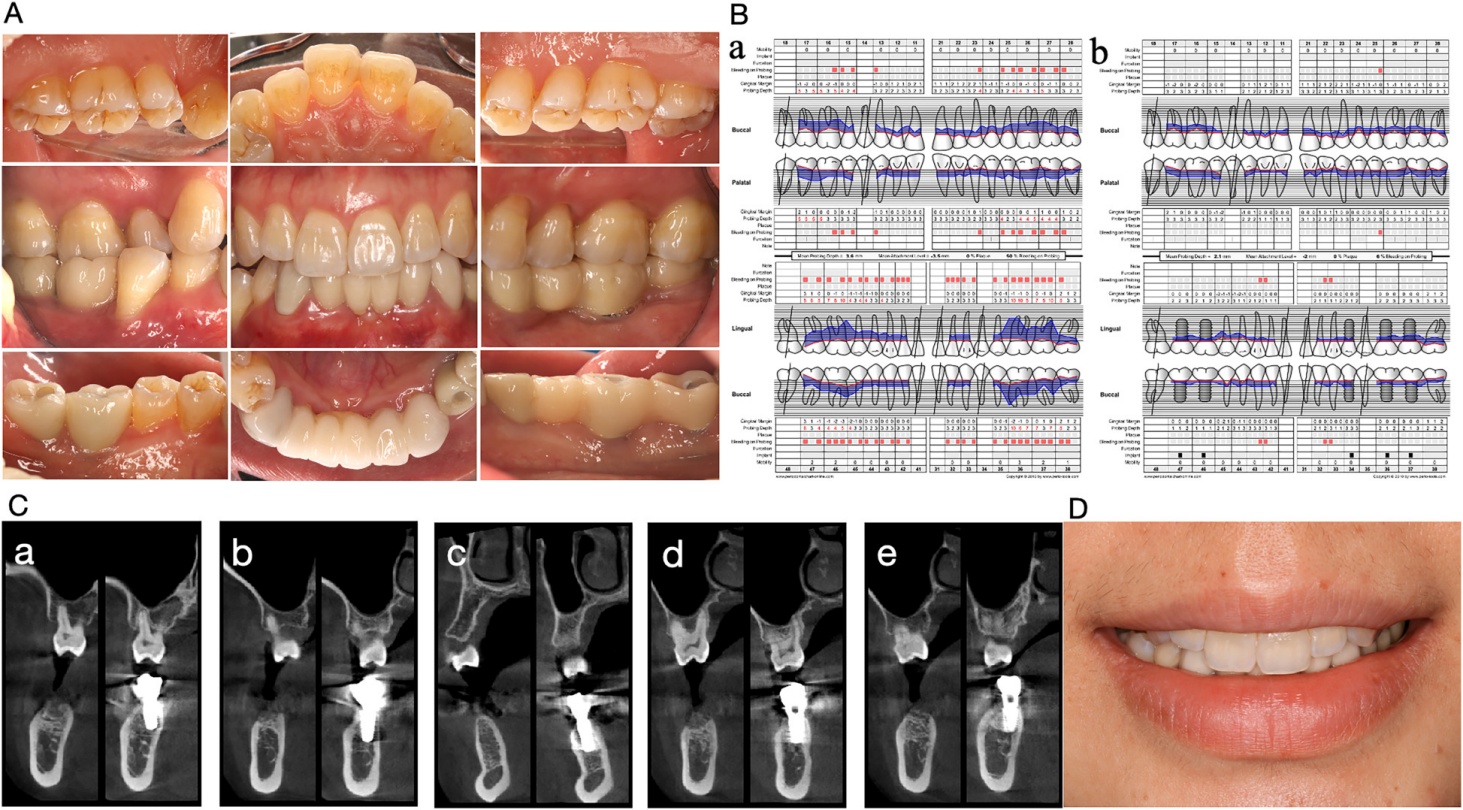
Oral condition of the Case 1 patient two years after implant restoration. **(A)** Intraoral photo. **(B)** Comparison of periodontal parameters. (a) Initial visit. (b) Two years after implant restoration. **(C)** CBCT comparison before and two years after implantation. From (a) to (e), the mandibular implants from right to left in case1 patients are shown before and two years after implantation. Implants were tightly integrated with the alveolar bone and the bone height was stable. **(D)** Frontal smiling photo.

Currently, this patient has been undergoing implant restoration for two years. The periodontal condition remains stable, and no abnormalities in the implant have been detected. Afterward, she accepted periodontal supportive therapy. Figure 2 shows her oral condition at the 2-year follow-up for dental rehabilitation.

## Case 2

The patient is the son of case 1. A 23-month-old boy had lost tooth 71 while playing at home, without any falls or hitting hard objects, and tooth 81 was slightly loose. He was delivered at full term via vaginal delivery, with a birth weight of 3.9 kg and a height of 51 cm. At his clinic visit, he weighed 12 kg and measured 88 cm in height. According to China's Growth Standards for Children Under Seven Years Old released in 2023, his growth was in the 50th percentile of the normal range.

The crown morphology, gingiva, and occlusion of his remaining teeth showed no abnormalities. The root length of tooth 71 matched the boy's age, with a wide pulp cavity, pitted resorption in the apical third of the root, and incomplete closure of the apical foramen.

Because non-traumatic tooth loss occurred at an age of less than 2 years without other abnormalities is extremely rare and the child's mother also has a history of tooth loss in age 15, a suspicion of HPP raised and a blood test was initiated for further evaluation. The results showed a serum alkaline phosphatase level of 64 U/L (reference range: 128–432 U/L), a serum urea level of 7.0 mmol/L (reference range: 2.3–6.7 mmol/L), a high-density lipoprotein cholesterol (HDL-C) level of 1.60 mmol/L (elevated ≥1.55 mmol/L), a serum sodium level of 135.6 mmol/L (reference range: 137–147 mmol/L), and a serum inorganic phosphate level of 2.20 mmol/L (reference range: 1.45–2.1 mmol/L). All other trace elements, electrolytes, vitamin D, liver function, kidney function, complete blood count, blood glucose levels, lipid profile, and thyroid function tests were within normal ranges. The above examination results are in line with our suspicion.

**Figure 3:**
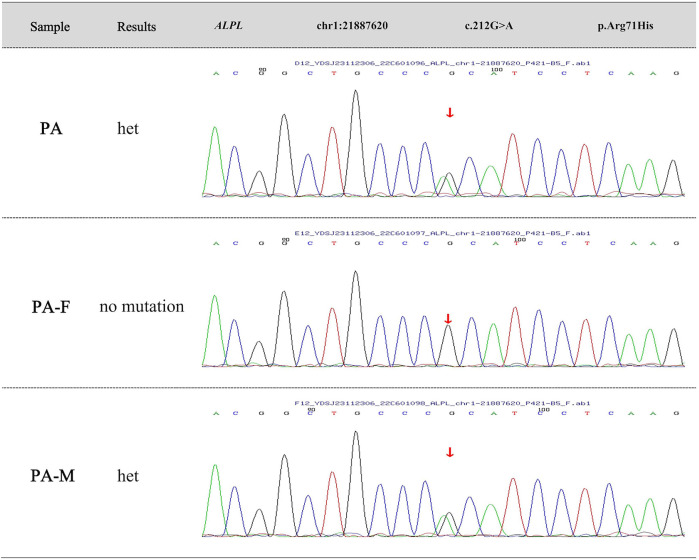
Family genetic mutation analysis. The genetic testing results reveal a heterozygous mutation at chromosome position chr1:21887620 in the ALPL gene, where nucleotide G is changed to A at position 212 (c.212G>A), resulting in an amino acid change from arginine to histidine at position 71 (p.Arg71His). The variation in ALPL gene originates from the affected child's mother.

Further, we suggest that they undergo gene sequencing analysis. High-throughput sequencing was performed on the child's sample and Sanger validation on the parents' samples. The genetic testing results revealed a heterozygous mutation at chromosome position chr1:21887620 in the *ALPL* gene, where nucleotide G is changed to A at position 212 (c.212G>A), resulting in an amino acid change from arginine to histidine at position 71 (p.Arg71His). According to “ACMG Standards and Guidelines for Clinical Sequencing” published by ACMG—a leading authority in medical genetics and genomics, this variant is preliminarily classified as “Pathogenic” based on PS3 + PS4 + PM2_Supporting + PM5 criteria. The variation in *ALPL* gene originated from his mother and the pedigree analysis results are shown in Figure 3. Ultimately, this mother received a definitive diagnosis of HPP after a decade-long delay.

Subsequently, this mother also underwent a blood test. The results revealed that her serum alkaline phosphatase level was 11 U/L (reference range: 35–100 U/L), and serum inorganic phosphate level was 1.79 mmol/L (reference range: 0.85–1.51 mmol/L).

This disease is caused by a heterozygous *ALPL* c.212G>A (p.Arg71His) variation, which may indicate autosomal dominant inheritance. However, since whole exome sequencing does not detect intron or promoter regions, the father could potentially be a carrier, suggesting an autosomal recessive pattern might also be possible. Thus, it is recommended: (1) Genetic counseling before planning pregnancy; (2) Preimplantation genetic testing; (3) Prenatal diagnosis to verify the fetal genotype once naturally conceived.

**Figure 4:**
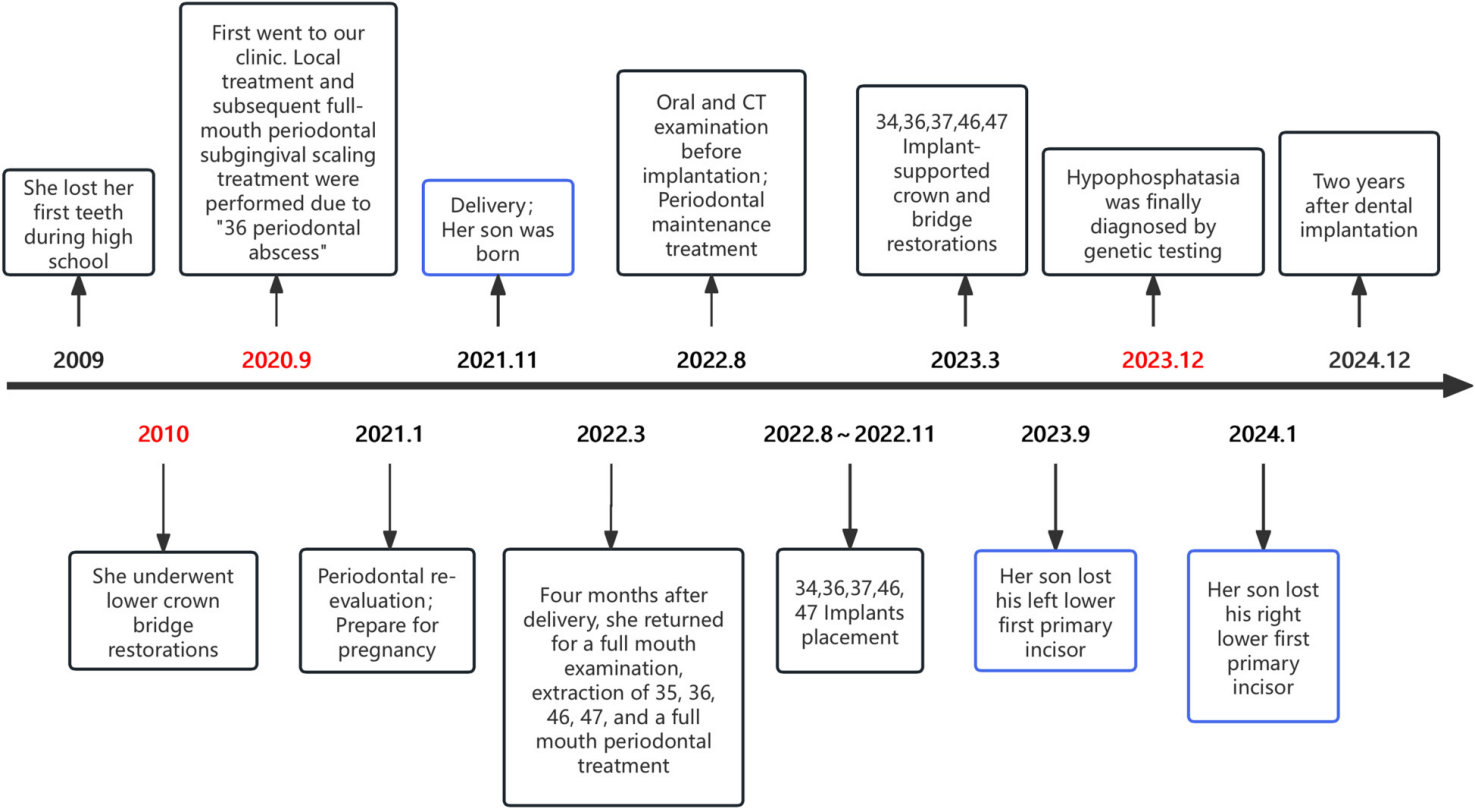
Timeline of diagnosis and treatment of these two cases.

After examination by a pediatrician, the child accepted regular physical and dental check-ups every six months. Tooth number 81 fell out three months later. The parents were instructed to maintain good oral hygiene for the child, prevent him from accidentally bumping or knocking tooth, and monitor any loosening of his teeth. The timeline of diagnosis and treatment for these two cases is shown in Figure 4.

## Discussion

### Mechanism of tooth loss in HPP

In HPP patients, early tooth loss results from developmental cementum aplasia or hypoplasia, leading to poor cementum-PDL attachment. Compared to wild-type mice, *Alpl^−/−^* mice exhibited osteoporosis and lacked an acellular cementum layer (8). This weak connection caused tearing during chewing. Immunolocalization analysis revealed the destruction of bone sialoprotein (BSP) and osteopontin (OPN), markers for dental mineralization, in *Alpl^−/−^* mice. Pyrophosphate accumulation reduced the thickness and adhesion of acellular cementum to dentin, while the apical region was less affected (9). Histopathological examination showed almost complete absence of acellular cementum and potential lack of cellular cementum. The periodontal ligament collagen fibers did not connect to the tooth root via Sharpey's fibers, separating from exposed dentin by a non-collagenous fiber layer. Reduced periodontal ligament coverage allowed bacterial plaque to extend to the bottom of periodontal pockets (10), consistent with our findings.

### Diagnosis criteria of pediatric and adolescent HPP

Recently, a consensus has been reached by the HPP International Working Group, composed of multidisciplinary experts from Europe, on the diagnostic criteria for pediatric and adolescent HPP (11). A prerequisite for the diagnosis of HPP is low ALP, excluding other conditions that cause low ALP. In addition, the diagnosis of HPP requires either two major criteria or one major and two minor criteria. In this report, the boy met two major criteria.

Based on the items in the consensus criteria (11), genetic testing is necessary for diagnosing genetic defects and diseases. However, the absence of *ALPL* mutations should not rule out a diagnosis of HPP because genetic studies of *ALPL* only sequence the coding region (exons) without considering the promoter or intronic regions (12). Some scholars pointed out that it was unnecessary to do genetic testing when clinical phenotype, radiology, and laboratory examinations were sufficient for diagnosis. However, genetic testing is crucial for documenting inheritance patterns, assessing recurrence risks, and prenatal diagnosis (13, 14).

As for the classification of HPP, the current classification methods are simple and useful, however, they fail to reflect the continuity of disease phenotypes (15). Patients who were unaffected during childhood may also develop rheumatic diseases and severe impairments due to bone metabolism conditions with age. The two key factors of HPP are its persistence and evolution throughout the entire lifespan. Therefore, their health changes should still be monitored throughout their lives and be provided with necessary and timely assistance.

Other possible causes of decreased serum alkaline phosphatase include diseases (hypoparathyroidism, hypothyroidism, renal osteodystrophy, achondroplasia, Wilson's disease, myeloma); drugs (antiresorptive agents, vitamin D excess, chemotherapy); miscellaneous [severe illness, major surgery or major trauma, massive transfusions; nutritional deficiencies (protein, calorie, zinc, folate, magnesium, vitamin B6, vitamin B12)].

Other reasons may lead to early loss of deciduous teeth, including external trauma, systemic diseases (such as neutrophil defects, aplastic anemia, Papillon-Lefevre syndrome, Chediak-Higashi syndrome, leucocyte adhesion deficiency, acatalasia and vitamin C deficiency), and diseases that damage the supporting tissues of teeth (such as Langerhans' cell histiocytosis and Ehlers-Danlos syndrome) (16, 17), which need differential diagnosis.

By contrast, other conditions present divergent systemic signatures: Papillon-Lefevre syndrome features palmoplantar hyperkeratosis; Chediak-Higashi syndrome and leucocyte adhesion deficiency involve immunodeficiency with recurrent pyogenic infections; vitamin C deficiency causes scorbutic manifestations (e.g., weakened capillary vessels, bleeding tendency and impaired wound healing); Langerhans' cell histiocytosis demonstrates osteolytic skull lesions and diabetes insipidus; and Ehlers-Danlos syndrome typically presents with joint hypermobility, skin hyperextensibility, and vascular fragility (18, 19). Importantly, serum ALP levels are normal or elevated in these conditions, unlike the ALP deficiency seen in HPP.

### Methods for screening suspected HPP in dentistry

Both perinatal lethal and benign prenatal forms of HPP can be confirmed through prenatal ultrasound examination, postnatal biochemical and genetic testing either in the uterus or immediately after birth. Patients with HPP in a dental clinic often present with early tooth loss as the main symptom. For children, especially under 3 years old, premature non-traumatic tooth loss should raise our attention. The cause of tooth loss (trauma, tooth replacement, caries, or unknown reasons) should be considered. It is important to carefully inquire about the parents' medical history, and refer the patient to a pediatrician in orthopedics or endocrinology for evaluation if necessary. Even if the parents do not show signs of early tooth loss, the relatives may have other symptoms of HPP, such as a history of fractures (20). Further testing should include serum ALP measurement or urinary ALP substrate detection. The reference values for ALP vary according to age and gender (21). It has been reported that the cases with normal blood ALP were confirmed through histological analysis of shed teeth tissue (17). Recently, a dental diagnostic system for HPP has been established in Japan with an additional oral screening project at 1.5 years and 3 years old through assessing early tooth loss history in primary teeth (22). Mild HPP is often misdiagnosed or diagnosed late, suggesting its incidence may be higher than expected. Perhaps we can consider adding “tooth loss” as an option in the oral exams during routine check-ups for children before they enter kindergarten, and uniformly report this data to enable further evaluation of these children. For people who undergo oral examinations during pregnancy preparation, if there is a situation of premature tooth loss (not due to caries, trauma, or extraction of wisdom teeth), it is recommended to add a serum ALP test for further evaluation.

When the patient has unclear systemic symptoms or an ambiguous medical history, it can easily be confused with familial aggregation-related “aggressive periodontitis” (1999 classification system for periodontal diseases). Compared to chronic periodontitis, aggressive periodontitis is described as an early-onset, rapidly progressing, periodontal destruction that does not correspond to the amount of plaque and exhibits a familial clustering pattern. The 2018 classification system for periodontal diseases abolished the diagnosis of “aggressive periodontitis” and replaced it with staging and grading descriptions for periodontitis diagnosis. However, the patient who exhibits similar features to “aggressive periodontitis” in clinical practice requires awareness: could it possibly be HPP?

In our cases, the female patient experienced a decade-long diagnostic delay and was misdiagnosed during her first visit in high school and at our clinic, until her son developed symptoms. The reasons for the misdiagnosis and delayed diagnosis of this case may be the incomplete family history of the patient, the oral examination being similar to periodontitis, the fact that the serum alkaline phosphatase level has never been examined, and the doctor's lack of awareness of HPP. Other studies have also found that 75% of patients with HPP were diagnosed as periodontitis only in their initial dental presentations, highlighting that it is essential to improve awareness of early signs of HPP among dentists and primary healthcare providers (2). These aspects should be noted for patients with early missing teeth such as the age, the cause of tooth loss, history of premature loss of primary teeth, bone fractures, bone pain, or family history of tooth loss. Reviewing the patient's oral x-rays or CBCT scans may provide some assistance in assessing bone density. Dual-energy x-ray absorptiometry (DXA) is considered the gold standard for diagnosing osteoporosis, which can support the diagnosis of HPP, although it is not necessary. Misdiagnosing HPP as “osteoporosis” and treating it with bisphosphonates can increase the risk of fractures in HPP patients (23). If in doubt, serum ALP or urinary ALP substrate can be further tested. For both children and adults, genetic testing is recommended for diagnosis when necessary.

### Dental management for HPP

Poor oral hygiene exacerbates the inflammation of the gums, leading to further absorption of alveolar bone and loosening of teeth. Therefore, effective initial therapy, periodontal surgery and supportive periodontal care, and proper oral hygiene instruction are crucial. Preventive protocols include good oral hygiene habits, using soft-bristled toothbrush and fluoride toothpaste, dental floss or interdental brushes, as well as avoiding other risk factors for periodontitis such as smoking (24). Regular follow-up visits for good periodontal maintenance are also essential.

Tooth loss causes difficulty in chewing, swallowing, aesthetics, and speech pronunciation (25). The use of partial dentures is necessary for the missing primary teeth in children, as they are in the process of growth and development. Some scholars demonstrated that partial denture for primary tooth loss should be done as early as possible when a child is around 3 years old and able to tolerate impression materials (22). For teenagers with severe HPP, limited reports have been demonstrated that traditional removable dentures, overdenture, and precision attachment were used due to multiple missing teeth, poor alveolar bone condition, or intellectual disabilities (26, 27). However, no long-term follow-up was observed, thus, it is hard to evaluate the prognosis.

In terms of implant treatment, one HPP patient with full mouth dental implants has been followed up for 7 years with stable implants, stable alveolar bone levels, and healthy gingiva (28). Successful implant cases have been followed up for a maximum of 13 years. Among seven implants, one failed due to a lack of initial stability (29). Also, although reduced alkaline phosphatase levels can affect bone mineralization, in our case, the female patient's dental implants and periodontal tissues remained stable after 2 years.

For childhood HPP, adult HPP, and odontohypophosphatasia, the frequency of dental reexaminations should be increased (18). Both adult HPP and odontohypophosphatasia may require multidisciplinary treatments, including periodontal, prosthodontic, and orthodontic therapies (30). Efforts should focus on preventing accompanying conditions like dental caries and erosion to avoid worsening the primary disease. Early oral rehabilitation planning is critical, with full-mouth functional reconstruction serving as the core of lifelong oral management (31). The goals are to relieve symptoms, restore function, and improve aesthetics.

A multidisciplinary and personalized approach should be planned as dental issues in these patients may involve almost all branches of dentistry. A coordinated and collaborative team may consist of doctors and nurses from various specialties, including pediatrics, orthopedics, endocrinology, dentistry, psychology, neurosurgery, nutritionists, and reproductive genetics experts (32). It enables seamless collaboration when dealing with patients suffering from these types of diseases and forms a treatment network.

### Enzyme replacement therapy (ERT) on oral health

Asfotase alfa (Strensiq®; Alexion Pharmaceuticals Inc, New Haven, CT, USA) is a bone-targeted recombinant TNSALP replacement drug, which is currently the only approved treatment for HPP. It can replace the activity of TNSALP and reduce substrate accumulation to improve bone mineralization. In 2015, (Food and Drug Administration) FDA approved this medication for the treatment of perinatal-, infantile-, and juvenile-onset HPP, with benefits observed in lung function, calcium homeostasis, skeletal health, and survival rates in infants and children (adolescents) with HPP. This drug has been approved for use in the United States, Canada, Europe, and Japan but has not yet been approved in China.

It is generally accepted that children with only the odontoHPP form are not suitable candidates for ERT. There is no evidence that ERT can improve dental outcomes in children with odontoHPP, and prevent periodontal disease progression in adults with HPP (33). The necessity and effectiveness of ERT in adults with mild HPP requires further investigation (34).

The health of both the mother and child will be monitored for accepting long-term multidisciplinary care. If the ERT drug can be introduced in the future, it will be applied based on the guidelines and the condition of their teeth after medication will be observed.

### Implications for clinical practice

Pediatric dentists, periodontists, and oral health care professionals lack awareness of this rare disease. Oral clinicians need to be vigilant about “non-traumatic premature tooth loss”, carefully inquire about medical history, and make a differential diagnosis based on clinical and laboratory examinations. Early identification of HPP patients may provide them with life-changing treatment options and contribute more evidence-based medicine for HPP management.

## Data Availability

The original contributions presented in the study are included in the article, further inquiries can be directed to the corresponding authors.
